# Evaluating CPR training: simulation vs. webinars for Iranian emergency medical technicians during COVID-19

**DOI:** 10.1186/s12873-024-00967-2

**Published:** 2024-03-18

**Authors:** Shoaib Sarboozi-Hosseinabadi, Gholamreza Sharifzadeh, Seyed Mohammadreza Hosseini

**Affiliations:** 1 Department of Emergency Nursing, School of Nursing and Midwifery, Birjand University of Medical Sciences, Birjand, Iran; 2 Department of Epidemiology and Biostatistics, School of Health, Social Determinants of Health Research Center, Birjand University of Medical Sciences, Birjand, Iran

**Keywords:** Cardiopulmonary resuscitation, COVID-19, Simulation-based training, Educational webinar, Competency, Emergency medical technician

## Abstract

**Introduction:**

The high prevalence of COVID-19 and the necessity for social distancing have impacted medical training. On the one hand, the high mortality rate following the disease led the American Heart Association (AHA) to issue guidelines in October 2020 for performing cardiopulmonary resuscitation on patients diagnosed or suspected of having COVID-19. Various methods exist for teaching these guidelines. However, the use of many of these methods is greatly challenged due to the high risk of disease transmission. Moreover, the published guidelines emphasize protection against COVID-19 infection. The present study aims to compare the impact of two educational methods, educational webinars and simulations, on the competence of performing cardiopulmonary resuscitation during the COVID-19 epidemic.

**Methods:**

This semi-experimental study was conducted on 70 emergency medical technicians. A pre-test was administered to all participants, and then they were randomly assigned into two groups: an educational webinar group (35 people) and a simulation group (35 people). The educational webinar group received online training using Adobe Connect software version 2.6.9, while the simulation group received in-person training using a manikin simulator. The competence of performing cardiopulmonary resuscitation during the COVID-19 epidemic was compared between the two groups immediately after the training and again two months later. Data collection instruments utilized in this research included a demographic questionnaire and a competency questionnaire in performing CPR during the COVID-19 pandemic. The data were analyzed using SPSS software version 19 and statistical tests for comparison.

**Results:**

The results indicated that in both the educational webinar and simulation groups, the average competence score for performing cardiopulmonary resuscitation (CPR) at the three stages under investigation showed significant differences (*p* < 0.001). Additionally, in both groups under study, the average competence score for performing CPR immediately and two months after training was significantly higher compared to before the training (*p* < 0.001); however, two months after training, it was significantly lower compared to immediately after the training (*p* < 0.001).

**Conclusions:**

Based on the results obtained from the current research, both educational methods (educational webinar and simulation) had a significant effect on the competence of performing CPR during the COVID-19 epidemic and were equally effective. Moreover, the recall test results (two months later) showed a decrease in the competence of performing CPR during the COVID-19 epidemic in both training methods (webinar and simulation), indicating the need for periodic CPR training.

**Supplementary Information:**

The online version contains supplementary material available at 10.1186/s12873-024-00967-2.

## Introduction

In late December 2019, reports emerged of an outbreak of a type of pneumonia with viral characteristics in the city of Wuhan, China [1–3]. The World Health Organization dubbed this incessantly spreading unknown disease COVID-19. This virus was highly contagious, capable of transmitting to others even before the onset of symptoms, posing a high risk of mortality, especially among the elderly and those with underlying health conditions [4–6].

To date, the COVID-19 pandemic has led to over 697 million infections and 6 million deaths worldwide [7]. According to statistics, the mortality rate among hospitalized patients has exceeded 20%, and in severe cases, it has approached close to 40%. Given these significant figures, the American Heart Association has published new guidelines for cardiopulmonary resuscitation (CPR) in patients suspected or confirmed to be infected with this virus [8, 9].

In the approach to CPR, the increased risk of aerosol generation from resuscitative processes, such as intubation and chest compressions, has compromised the competence and safety of resuscitation operations. These circumstances can lead to hesitancy and delay in administering CPR [10–12].

In response to these challenges, the American Heart Association provided interim guidelines in October 2020 to facilitate the resuscitators’ responses to cases of cardiac arrest suspected or confirmed to be due to COVID-19 [13]. These updated guidelines, based on specialized knowledge and adaptation to current circumstances, emphasize the importance of training this guidance to medical and nursing staff [14–16].

The American Heart Association has continually stressed the importance of practical CPR training methods, such as simulations with advanced manikins and providing precise feedback. These types of training significantly improve the accuracy and skill of healthcare professionals in resuscitation [16–18].

However, the COVID-19 pandemic has put these traditional training methods in the face of unusual and serious challenges. The risk of disease transmission in face-to-face sessions and concerns raised by health care workers have necessitated a reevaluation of educational methods and an increased use of digital technologies like webinars [19, 20]. Research by Kou and colleagues showed that advanced life support skills in the simulation group were much higher than in the traditional training group [21]. Similarly, Saiedi and colleagues demonstrated that in the simulation method, the average student scores were significantly higher than those in the traditional group [22].

Given the importance of CPR in patients with COVID-19 and the limited research in Iran, this study sought to assess the effectiveness of CPR training through educational webinars compared with simulation, and their impact on CPR competence. In doing so, the study aimed to identify potential alternative training methods during the COVID-19 pandemic, if successful.

## Methods

### Study design

The present study was a semi-experimental investigation conducted on 70 pre-hospital emergency medical technicians in the city of Khaf in the year 2021.

The study was classified as semi-experimental due to its use of both pre-test and post-test measurements, as well as the comparison of outcomes between the two different educational methods, making it appropriate to characterize the study as semiexperimental.

### Participants

Eligibility criteria for this study included being an active medical emergency technician at the Emergency Medical Services Unit 115, having at least an associate degree in medical emergency, access to the Internet and social networks, no participation in advanced CPR training courses for COVID-19 in the past six months, agreement to participate in the study and signing a consent form, and having suitable physical health to perform study activities. Exclusion criteria included unwillingness to continue participating at any stage of the study and absence from training and operational sessions.

### Outcomes

Data collection instruments utilized in this research included a demographic questionnaire and a competency questionnaire in performing CPR during the COVID-19 pandemic. We developed the questionnaires specifically for this research, ensuring that they are tailored to the unique aims and contexts of our investigation (supplementary file 1).

The demographic information questionnaire comprised five questions regarding age, work experience, marital status, education, and employment status. It was filled out through interviews with the participants. The demographical questionnaire was researcher-generated, and its validity was established through content validity. For this purpose, corrective feedback from ten faculty members of the Nursing-Midwifery School at Birjand University of Medical Sciences was employed. Participants completed the demographic information questionnaire at the beginning of the study.

The CPR competency questionnaire for patients with/ suspected of COVID-19 was also researcher-generated. This questionnaire was based on the 2020 American Heart Association guidelines and included 20 questions about the basic life support (BLS) principles. Topics covered were recognition of signs of cardiac-respiratory arrest, assessment of victim’s responsiveness and vital signs, proper positioning, calling for assistance, determination of the number and depth of chest compressions during cardiac massage, initial airway management, performing artificial respiration, and use of AED (Automated External Defibrillator). The scores of this questionnaire ranged from zero to twenty points, with higher scores indicating higher competency of pre-hospital emergency personnel in adult BLS. For validity confirmation of the questionnaire, it was submitted to ten faculty members of Birjand University of Medical Sciences and for reliability determination, it was presented to 10 non-participating emergency medical technicians, obtaining a Cronbach’s alpha coefficient of 0.84.

### Sample size

Based on the study of Rabiei Pour et al. [23], and the average learning score of students in the virtual group (6.85 ± 1.62) vs. the traditional group (8.95 ± 3.24), with a test power of 90%, a significance level of 0.05, and a sample size of 31 individuals per group was determined, considering a drop-out probability of 10%, the current study included 35 individuals in each group [23].

$$ n=\frac{7.8 ({1.62}^{2}+{2.34}^{2})}{{(6/85-8/95)}^{2}}\approx 31$$

### Data collection

#### Participants selection

Participants were selected from a diverse range of urban and roadside emergency bases in Khaf. Through the use of SPSS software, they were randomly assigned to either the educational webinar group or the simulation group, utilizing a pattern generated by the SPSS software.

#### Simulation group training

The simulation group underwent comprehensive training in line with 2020 American Heart Association guidelines for resuscitation courses. The training materials used in both the simulation and educational webinar methods were indistinguishable. They included protocols for managing patients experiencing suspected COVID-19 cardiac-respiratory arrest, guidelines for preventing emergency personnel from contracting COVID-19 during cardiac procedures and intubation, detailed instruction on the use of protective equipment, and adherence to standard precautions during CPR as outlined by the American Heart Association for patients suspected of having COVID-19. In the simulation group, the training took place over the course of one day, spanning 8 h (morning and afternoon), and was followed by a post-test.

#### Educational webinar group training

For those in the educational webinar group, the exact training materials taught in the simulation-based group were made available online through the Adobe Connect application version 2.6.9. Participants accessed the virtual classroom via a link provided by the course instructor and engaged in an 8-hour training session, paralleling the duration and structure of the simulation method. Practical training was facilitated through the use of instructional videos containing the training materials, which were uploaded for participants to view. Additionally, scenarios were presented by the class instructor for discussion among participants. Within the software’s interface, attendees were actively engaged in responding to questions, and the instructor enabled the use of microphones for participants to engage in Q&A sessions. At the conclusion of the online class, all participants in the webinar completed an electronic questionnaire that had been prepared in advance.

#### Follow-up re-test

It should be noted that a follow-up re-test was conducted electronically two months after the conclusion of the educational intervention for both training groups, with participants receiving a link to access the questionnaire.

### Statistical analysis

The questionnaire scores assessing CPR competency during the COVID-19 pandemic, before and after training, and two months post-training were compared between the two groups using SPSS version 19. Demographic data were analyzed using the chi-square test. To determine and compare the mean competency scores for performing CPR intragroup, the ANOVA test was used due to the normal distribution of data; for intergroup comparison of mean competency scores, the independent t-test was used. A significance level of less than 0.05 was considered.

## Results

The demographic analysis revealed no significant differences between the Simulation Method group (*n* = 35) and the Training Webinar group (*n* = 35), with respect to mean age (27.17 ± 4.71 vs. 27.40 ± 4.02, *P* = 0.83), work experience (4.74 ± 4.12 vs. 4.77 ± 3.49, *P* = 0.98), marital status (single: 31.4% vs. 40%, married: 68.6% vs. 60%, *P* = 0.45), educational background (Associate’s degree: 42.9% vs. 51.4%, Bachelor’s degree: 57.1% vs. 48.6%, *P* = 0.47), and employment status (fixed: 54.3% vs. 57.1%, temporarily: 45.7% vs. 42.9%, *P* = 0.81). These findings indicate a comparable background among participants of both groups, as detailed in Table 1.

**Table 1:** Demographic variables of the simulation method and training webinar

Variable	Group		*P* value
	Simulation method (35)	Training webinar (35)	
Age (mean ± SD)	27.17 ± 4.71	27.40 ± 4.02	*P = 0.83
Work experience (mean ± SD)	4.74 ± 4.12	4.77 ± 3.49	*P = 0.98
Marital status n (%)			
Single	11 (31.4)	14 (40)	**P = 0.45
Married	24 (68.6)	21 (60)	
Education n (%)			
Associate’s degree	15 (42.9)	18 (51.4)	**P = 0.47
Bachelor’s degree	20 (57.1)	17 (48.6)	
Employment status n (%)			
Fixed	19 (54.3)	20 (57.1)	**P = 0.81
Temporarily	16 (45.7)	15 (42.9)	

*Independent T-test

**Chi-square test

Based on the findings in Table 2, there was no significant difference between the two groups—educational webinar and simulation—in terms of the average competency score for performing cardiopulmonary resuscitation (CPR) before, immediately after, and two months after the training (with p-values respectively at 0.84, 0.28, and 0.78). Additionally, the results indicated that in both the educational webinar and simulation groups, there was a significant difference in the average competency score for performing CPR across the three stages examined (*p* < 0.001) (Table 2).

**Table 2:** Comparison of two groups of simulation method and training webinar

Variable		Group		*P*
		Simulation method (35)	Training webinar (35)	
CPR competency (Mean ± SD)	Before training	10.43 ± 0.3	10.29 ± 2.98	*0.84
	After training	16.20 ± 1.81	16.69 ± 1.92	*0.28
	2 months after training	12.77 ± 2.73	12.97 ± 3.26	*0.78
P		**P < 0.001	**P < 0.001	

*Independent T-test

**ANOVA

## Discussion

The study’s findings indicated that the simulation training group showed a significant increase in competency for performing cardiopulmonary resuscitation (CPR) by emergency medical technicians during the COVID-19 pandemic compared to before the training. Previous studies by Al-Hadid et al., Kou and colleagues, and Saiedi et al. also demonstrated significant increases in knowledge and skill for CPR performance in groups undergoing simulation training compared to traditional training methods [24–26].

The present study also found that in the webinar training method, the average competency score for performing CPR during the COVID-19 pandemic by emergency medical technicians significantly increased after training compared to before. In line with this, Rogers et al., in 2013, in a study on the effectiveness of online CPR training for rural and remote nurses, showed a marked improvement in CPR competency scores after training [27]. Furthermore, Cason and colleagues evaluated the impact of online and traditional training on CPR skills and found that participants in online courses performed and excelled with better skills compared to the traditional method [28]. Tobias et al. also demonstrated in their study, “The Impact of Online Basic Life Support Training,” that online courses are an effective method for teaching and learning CPR skills [29]. Another study by Ann and colleagues on “The Impact of a Mobile Phone Reminder Video on CPR and AED Skills” found that the acquired skills in performing CPR and using an automated external defibrillator were significantly higher compared to the control group. Even after a three-month recall test, the CPR performance of the trained group surpassed that of the control group [30].

Another finding of the study was that the average changes in CPR competency scores during the COVID-19 pandemic by emergency medical technicians two months after training in the webinar training group did not significantly differ from the simulation training method, and both methods were equally effective. Similarly, Cardong and colleagues compared the impact of computer-based versus mannequin with audio feedback on CPR skills and concluded that there was no difference in chest compression rates between the two courses. However, participants in the mannequin method with audio feedback achieved more compressions with sufficient depth and correct hand positioning [31]. In a 2007 study, Tatal et al. compared the conventional, web-based, and simulation training methods and found that both the simulation and web-based methods were equally effective in enhancing skills and competencies [32]. Similarly, Bahadorani and colleagues assessed the effectiveness of three teaching modes in online, face-to-face, and blended learning, showing that there was no significant statistical difference in knowledge, skills, and satisfaction among the three groups, indicating that all three instructional methods were equally effective [33]. Senna and colleagues evaluated the comparison of a video-based online course versus a serious game on CPR and showed that knowledge and skill scores improved equally in both groups [34]. Furthermore, Khoshnevisfar et al. investigated the impact of electronic versus traditional teaching methods on nurses’ awareness, skills, and satisfaction, demonstrating that both educational approaches led to an increase in these areas for the nurses [35], aligning with the results of the current research. Another study by Nass et al. on “The Impact of Face-to-Face versus Virtual Reality Training on the Quality of Cardiopulmonary Resuscitation” showed that chest compression quality was lower in the virtual method compared to face-to-face. This study was not aligned with he current research and the discrepancy might be explained by the fact that in their study, the virtual training was only 20 min long, while in our study, the online training was conducted over an eight-hour span which could contribute to a greater impact [36].

Additionally, the average changes in post-training CPR competency scores during the COVID-19 pandemic two months later did not significantly differ between the webinar training group and the simulation training method, with both training methods showing a decrease in CPR competency. In line with this, Tatal and colleagues’ recall test results indicated a decrease in competency scores but it was not significant [32]. This could be due to the fact that, in that study, practice and simulation were carried out on patients which could lead to better information retention. Moreover, in that study, participants were respiratory care practitioners who interacted with these patients daily in intensive care units for two months until the recall test, unlike in our study where pre-hospital emergency personnel had much less exposure to performing CPR on COVID-19 patients over the same period. In the study by Ann et al. mentioned earlier, recall test results showed that the group with training retained skills better than the control group after three months [30], which could be because the training involved basic CPR and automated external defibrillator instruction, which is easier to remember and recall than advanced CPR during the COVID-19 epidemic with new content. Also, the two-month post-test results of the study by Goodarzi et al. on the effectiveness of basic infant CPR training on nurses’ knowledge and performance indicated a decline in knowledge and performance levels [37], which aligns with our study’s findings.

The incorporation of interaction and collaborative problem-solving in educational methodologies significantly shapes the learning experience and the acquisition of practical skills [39–40]. In the realm of emergency medical training, where split-second decisions and precise execution can be a matter of life and death, the significance of such active learning strategies cannot be overstated [41]. Our study on online training for CPR performance stands out in its deliberate emphasis on fostering trainee-trainer interaction and collaborative problem-solving, distinguishing it from other studies that may have adopted more passive online training methods.

The unique feature of our study lies in its utilization of online training that goes beyond mere information dissemination. By facilitating forced trainee-trainer interaction and promoting collaborative problem-solving, our approach translates theoretical knowledge into practical decision-making and action. This dynamic learning environment not only nurtures a deeper understanding of CPR protocols but also cultivates the judgment and adaptability necessary for effective real-world application [42]. In stark contrast to the conventional approach of passive online learning, characterized by video-based instruction, our training method actively engages participants, eliciting discussions, problem-solving, and immediate feedback.

Moreover, by leveraging technology to facilitate this interaction and problem-solving in an online format, our study acknowledges the evolving landscape of education and professional development. The webinar training bridges the gap between traditional face-to-face instruction and remote learning, offering a viable and effective alternative for ongoing professional development, especially in the context of a global pandemic [43].

Based on the provided information, it appears that the study’s findings have demonstrated a significant increase in CPR competency among emergency medical technicians following online webinar training. This contrasts with the traditional passive online training methods often involving video-based instruction. The study appears to have strategically emphasized trainee-trainer interaction and collaborative problem-solving, setting it apart from other studies and underscoring the dynamic learning environment created by its training approach.

Among the limitations of this study were that 1- participants who did not have a smartphone could not benefit from this educational method and were excluded from the research; 2- due to maintaining social distancing among participants, it was not possible to assess CPR performance skills through Objective Structured Clinical Examination (OSCE), and a questionnaire was used for evaluation instead. Additionally, this research was conducted on medical emergency personnel, limiting the scope of the study population. Therefore, it is recommended that future studies be carried out on a broader range of healthcare staff and with a larger sample size to assess the impact of these educational methods. Furthermore, in light of the decrease in CPR competency scores during the recall test, periodic online training should be implemented for retraining healthcare personnel by educational authorities.

## Conclusion

Based on our research findings, both educational methods (webinar and simulation) significantly improved CPR competency during the COVID-19 epidemic. However, a decrease in competency was observed in both methods two months after training, highlighting the need for periodic CPR training. This decrease may be due to the limited exposure of medical personnel to CPR on COVID-19 patients and the novelty of CPR skills during the pandemic. As there was no significant statistical difference between the two methods, they can be integrated into future CPR training programs for healthcare workers, including those in remote areas.

### Electronic supplementary material

Below is the link to the electronic supplementary material.

Supplementary Material 1

## Data Availability

The datasets generated in the current study are available from the corresponding author upon reasonable request.

## Abbreviations

CPR: Cardiopulmonary resuscitation
BLS: Basic life support
AED: Automated external defibrillator
AHA: American Heart Association
