# Supplementary material for: Evaluating CPR training: simulation vs. webinars for Iranian emergency medical technicians during COVID-19
Source: BMC Emerg Med. 2024 Mar 18;24:46. doi: 10.1186/s12873-024-00967-2 (PMC10949631; doi:10.1186/s12873-024-00967-2)
Supplement: Supplementary file 1 — Supplementary Material 1 [file 12873_2024_967_MOESM1_ESM.docx]

| **demographic information questionnaire** | |
| --- | --- |
|  | Age |
|  | Marital status |
|  | Education |
|  | Employment Status |
|  | Work experience |
| Completion date............................. | |

**supplementary file 1**

**Competency questionnaire in performing CPR during the COVID-19 pandemic**

1. You come to the bedside of a patient who tested positive for Covid-19 four days ago and currently has no vital signs. You try to intubate and insert a supraglottic airway, but your efforts are unsuccessful. ?
   1. Active oxygenation of the mask with storage bag and HEPA filter
   2. Passive oxygenation with a one-way mask and covering the mask with a surgical mask
   3. Ventilation with BVM with HEPA filter and tight fitting of the mask to the face
   4. Ventilation with BVM with a one-way mask and firmly sticking the mask to the face
2. You are performing cardiac resuscitation on a 43-year-old man suffering from Covid-19, the capnograph attached to the end of the patient's tracheal tube shows the number 40. Which of the following would you do?
   1. Checking the patient's pulse
   2. Checking the patient's tracheal tube
   3. Continue CPR
   4. Hypoventilate the patient
3. After the intubation of a 63-year-old man suffering from covid-19 who suffered cardiorespiratory arrest; How do you perform the sequence of ventilation and cardiac massage?
4. One breath every 6 seconds by stopping chest compressions
5. One breath every 8 seconds by stopping chest compressions
6. One breath every 6 seconds without stopping to squeeze the rack
7. One breath every 8 seconds without stopping chest compressions
8. You arrive at the bedside of a 53-year-old woman. After the examination, you realize that the patient has no vital signs. The companions state that the patient has had cold symptoms for the past few days. You start cardiopulmonary resuscitation for the patient. You see the following rhythm in the monitor. What is your first action?


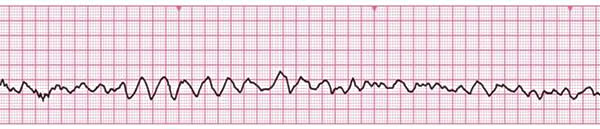


1. Passive oxygenation with BVM
2. Early intubation of the patient
3. Perform cardiac massage for 2 minutes
4. Shock discharge of 200 joules
5. Cardiac resuscitation is currently being performed on a 50-year-old patient who tested positive for Covid-19 10 days ago. After 2 minutes of Turing cardiac massage, you will notice the following rhythm; What is your first action?


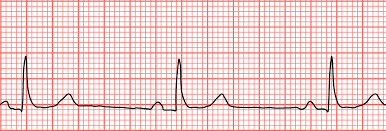


1. Checking the patient's pulse
2. Stopping cardiac massage and putting the patient in recovery mode
3. Giving two breaths to the patient
4. Intravenous atropine injection of 1 mg
5. At the beginning of basic cardiopulmonary resuscitation and before intubation in a 65-year-old patient suspected of having covid-19, what is not appropriate to do to prevent aerosol production during ventilation?
6. Massage along with ventilation with BVM connected to the HEPA filter and firmly sticking the mask on the face
7. Massage with passive oxygenation with a one-way mask and covering it with a surgical mas
8. Massage with active oxygenation with a mask with a storage bag
9. Continuous massage without ventilation with a surgical mask for the patient\
10. You appear at the bedside of a 60-year-old patient who has a history of hypertension and diabetes. The companions state that his covid-19 test was positive in the past few days. After the examination, you realize that the patient has no vital signs, what is your first action?
11. Wear personal protective equipment
12. Preparing for intubation
13. Start cardiac massage
14. Providing oxygen with a mask
15. You come to the bedside of a 45-year-old man who has been in the hospital for the past week due to Covid-19 and was discharged from the hospital two days ago. does not have vital signs in the examination; In order to intubate the patient, according to which sequence do you act?
16. Intubation, then placing a HEPA filter, checking the correctness of intubation, filling the cuff of the tracheal tube and ventilating the patient
17. Intubation, then filling the cuff of the tracheal tube, placing the HEPA filter, checking the correctness of intubation and ventilation of the patient
18. Intubation, then placing a HEPA filter, patient ventilation, checking the accuracy of intubation and filling the tracheal tube cuff
19. Intubation, then placing a HEPA filter, checking the accuracy of intubation, patient ventilation and filling the endotracheal tube cuff
20. You appear at the bedside of a patient who has had symptoms of Covid-19 for the past few days and currently has no vital signs. After connecting the monitoring device to him, you notice the following rhythm; Which of the following actions is not a priority?


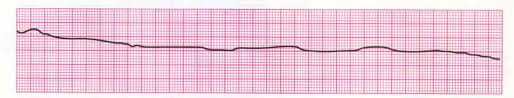


1. Intravenous epinephrine injection
2. Cardiac massage
3. Passive oxygenation
4. Patient defibrillation
5. Outside the work shift and without equipment, you come across a 6-year-old child who suffered a cardiac arrest. The child's mother states that her child had shortness of breath for about 1 hour before and was unconscious a few moments ago. What method do you use to give him breathing?
6. Due to the low probability of covid in children, it is safe to breathe mouth to mouth
7. We use family members to breathe the child.
8. By placing a cloth over the child's mouth, we perform mouth-to-mouth breathing for him
9. To maintain our safety, we wait for the emergency to start resuscitation
10. You are performing cardiac resuscitation on a 62-year-old man who is suffering from covid-19. In order to maximize the safety of personnel during resuscitation, which conditions do you not recommend for an advanced airway?
11. Intubation by video laryngoscope
12. Use of personnel with intubation skills in the first attempt
13. LMA insertion to ensure rapid insertion of an advanced airway
14. Using a surgical mask by a technician during patient intubation

You arrive at the bedside of a 6-year-old child who, according to his mother, has been suffering from covid-19 since a week ago and has had severe shortness of breath for half an hour and has suffered a loss of consciousness. In the examination, there are no vital signs and central cyanosis is evident.)

According to the above explanation, answer the next 3 questions.

1. Which of the following actions do you take to establish an advanced airway?
2. Use the most skilled person to perform intubation with a high probability of success
3. Using an uncuffed endotracheal tube if available
4. Use of pulse oximetry to confirm the position of tracheal tube
5. All of the above are correct
6. In order to increase the quality of cardiac resuscitation, the depth and number of cardiac massage according to which of the following items do you perform?
7. Compressing the chest with a depth of 1/2 or more of the anterior posterior diameter of the chest and the number of 100 to 120 times per minute.
8. Compressing the chest with a depth of 1/3 or more of the anterior-posterior diameter of the chest and 100 to 120 times per minute.
9. Compressing the chest with a depth of 1/2 or more of the anterior-posterior diameter of the chest and the number of 120 to 150 times per minute.
10. Compressing the chest with a depth of 1/3 or more of the anterior-posterior diameter of the chest and the number of 120 to 150 times per minute.
11. You arrive at the bedside of a 74-year-old patient who, according to his companions, tested positive for Covid-19 a week ago and had shortness of breath since last night. It is evident in the monitoring of the following rhythm, which of the following actions is the priority?
12. First, check the patient's pulse and if there is no pulse, defibrillate the patient with 200 joules
13. First, check the patient's pulse and if there is no pulse, cardioversion shock at the rate of 120 joules
14. Performing rapid defibrillation of the patient at the rate of 200 joules
15. Use of 200 joule synchronized shock

You appear at the bedside of a 54-year-old patient, after the examination you realize that the patient has no vital signs, you immediately start cardiac resuscitation measures. His wife states that the patient has been suffering from covid-19 since last week, and now he has severe shortness of breath and he was unconscious moments before.

According to the above explanation, answer the following two questions.

1. According to the above explanation, which of the following is correct for transferring the patient?
2. None of the family members and those who have been in contact with the suspicious person should ride in the ambulance.
3. If cardiac resuscitation is performed and ROSC is not achieved, the patient should be transferred to the nearest hospital.
4. During the transfer of the patient to the hospital for ventilation, it is mandatory to use the fan ambulance exhaust intermittently.
5. Items b and c are correct.
6. In the CPR process of the above patient, when are we allowed to stop CPR for evaluation?
7. If the heartbeat returns
8. After a minute
9. Once every two minutes
10. When replacing the regenerator
11. During the covid-19 epidemic, what actions should the emergency medical worker take to start basic resuscitation while walking in the neighborhood park with an adult who is suffering from cardiorespiratory arrest?
12. While wearing a mask, the paramedic starts performing CPR in the form of 30 massages and 2 breaths.
13. While wearing a mask, the rescuer should start continuous cardiac massage.
14. While holding a mask and placing the mask on the patient's face, the rescuer should start continuous heart massage.
15. The rescuer must wait for the emergency to arrive for his safety.

You are performing cardiac resuscitation procedures on a 45-year-old patient who has been hospitalized since a week ago due to shortness of breath caused by the Covid-19 disease and is currently suffering from cardiorespiratory arrest.

According to the above explanation, answer the following 3 questions.

1. Which conditions are safer in reviving the patient?
2. Resuscitation while the patient is intubated and connected to the ventilator using a HEPA filter
3. Resuscitation while the patient is intubated and connected to the Ambubag by a HEPA filter
4. Reviving using Ambobag with filter is fully capped
5. Resuscitation without ventilation and use of passive oxygenation
6. Which action is less likely to produce aerosols and airborne contamination?
7. Heart massage
8. Ventilation with BVM
9. shock discharge
10. Intubation
11. Which of the following is not correct regarding intubation of the above patient?
12. A closed suction system should be used to suction the patient.
13. Use high efficiency filters (HEPA) in the exhalation path.
14. Using a video laryngoscope for intubation is preferred over direct vision.
15. Intubation is not one of the procedures that produce aerosols, it is only ventilation that leads to the release of aerosols.
